# Disabling of ARC1 through CRISPR–Cas9 leads to a complete breakdown of self-incompatibility responses in *Brassica napus*

**DOI:** 10.1016/j.xplc.2022.100504

**Published:** 2022-12-14

**Authors:** Kumar Abhinandan, Neil M.N. Hickerson, Xingguo Lan, Marcus A. Samuel

**Affiliations:** 1 University of Calgary, Department of Biological Sciences, 2500 University Drive NW, Calgary, AB T2N 1N4, Canada; 2 20/20 Seed Labs Inc., Nisku, AB T9E 7N5, Canada; 3 Key Laboratory of Saline-Alkali Vegetation Ecology Restoration, Ministry of Education, College of Life Sciences, Northeast Forestry University, Harbin 150040, China

Dear Editor,

Many flowering plants utilize the self-incompatibility (SI) response as a genetic mechanism to prevent self-pollen from establishing on the stigmas, thereby promoting outcrossing and genetic diversity. In *Brassica* during SI, recognition of the pollen ligand SP11 by S-locus receptor kinase (SRK) results in activation of the E3 ligase ARM-Repeat-Containing protein (ARC1), which leads to proteasomal degradation of compatibility factors required for successful pollen acceptance. ARC1 was originally identified as an interactor of the SRK kinase domain and is highly expressed in mature stigmas (Gu et al., 1998). Antisense suppression of ARC1 resulted in partial breakdown of SI in the self-incompatible *Brassica napus* W1 line, establishing the role of ARC1 as a positive regulator of SI (Stone et al., 1999). This was further supported by RNAi-mediated suppression of ARC1 in the self-incompatible *A. lyrata*, which resulted in partial breakdown of the SI pathway (Indriolo et al., 2012). The observed partial compromise of SI in both *Brassica* and *Arabidopsis* suggested that either an alternative SI pathway or incomplete suppression of ARC1 could have resulted in the incomplete breakdown of SI. This question has remained unresolved. In this study, we created *ARC1* loss-of-function *B. napus* lines to demonstrate the central role of ARC1 in mediating the SI response.

Despite the partial breakdown of SI shown in two different systems, the role of ARC1 in the Brassicaceae SI response has been questioned for the past several decades. Stable co-expression of *Brassica SLG*, *SRK*, and *ARC1* was insufficient to confer the SI phenotype in compatible *Arabidopsis thaliana* (Bi et al., 2000). In another report, when *A. thaliana* plants were transformed to express *SRK*_*b*_ and *SCR*_*b*_ genes, a strong SI phenotype was observed in the absence of ARC1 (Kitashiba et al., 2011). *A. thaliana* plants that exhibit the SI phenotype through transformation of *SRK* and *SCR* alone show trends in heritability, developmental regulation, and intensity of SI response similar to those of self-incompatible *Brassica* cultivars (Nasrallah and Nasrallah, 2014). Although, evolutionarily, *Arabidopsis* that lacked a *bona fide* ARC1 ortholog could have re-purposed other E3 ligases to assume the role of ARC1, the question of how complete loss of ARC1 could influence SI in *Brassica* sp. remained unresolved.

To unequivocally examine the role of ARC1 during SI, we created *ARC1* loss-of-function *B. napus* using the CRISPR–Cas9 platform. In order to efficiently design specific targets for the CRISPR constructs, we retrieved available gene copies of *ARC1* or similar genes present in *B. napus* (allotetraploid from the A and C genomes of *B. rapa* and *B.* oleracea) (http://cbi.hzau.edu.cn/bnapus/index.php) (Song et al., 2020). After validation of the sequences across the published NCBI database, TOPO cloning was performed with *BnARC1-*specific cDNA amplification products obtained from an RNA pool of fully mature *B. napus* stigmas. Sanger sequencing of the clones revealed that *BnARC1* is a single-copy gene derived from *Brassica rapa* (A genome of *rapa*), whereas its homolog from the C genome (oleracea) was identified at low frequency and was most similar to *PUB17* family genes (Figure 1A). When the expression profiles of these two genes were assessed at various stages of stigma maturity, *ARC1* had significantly higher expression than *PUB17* and peaked at stigma maturity (Figure 1B).

**Figure 1:**
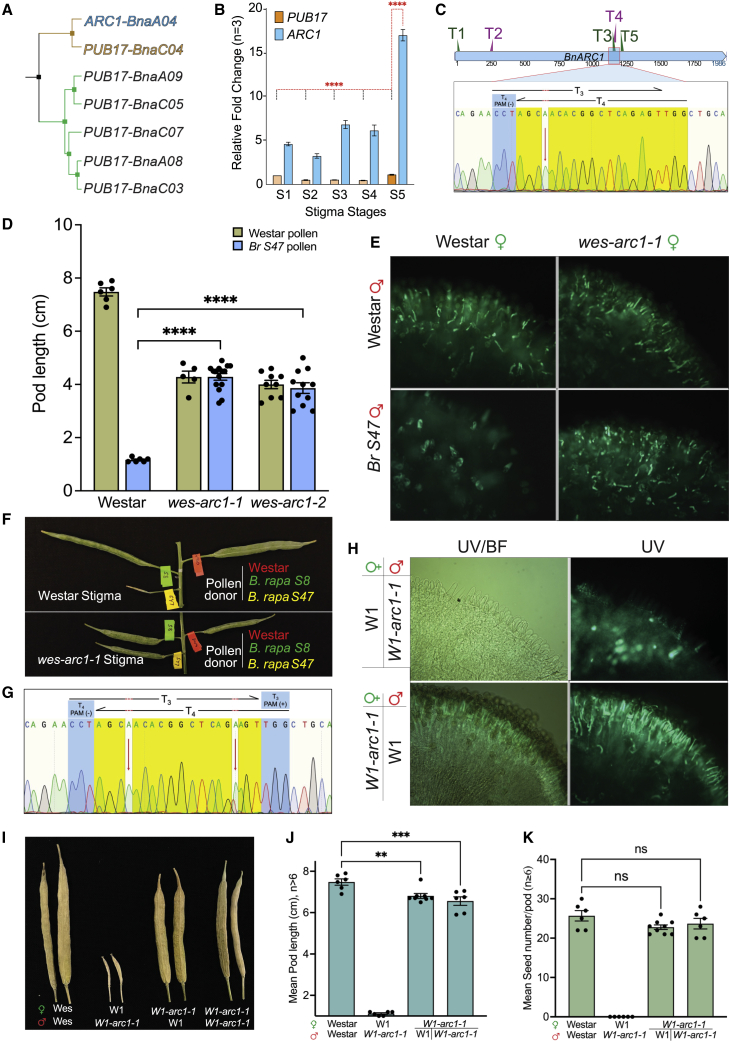
CRISPR–CAS9-mediated editing of *ARM-Repeat-Containing 1* protein (*ARC1*) leads to a complete breakdown of the self-incompatibility response in *Brassica napus*. **(A)***ARC1* and related sequences were retrieved from the *Brassica napus pangenome information resource* database. Highly divergent *PUB17* (*PUB17-BnaC03a*) is not shown in the cladogram. **(B)** Relative expression of *ARC1* and *PUB17* during stigma development was assessed by qPCR. The bars show the fold change in *BnPUB17* and *BnARC1* during stigma development. Error bars represent the standard error of the mean (±SEM). **(C)** Schematic diagram showing the location of the various guideRNA sites along with the chromatogram generated from Sanger sequencing of *BnARC1* from *wes-arc1-1.* Sequence spans the T_3_ and T_4_ target sites within the *BnARC1* coding region. The red arrow indicates the biallelic insertion of an “A” at precisely 3 bases from the T_4_ protospacer adjacent motif (PAM) sequence, indicating that the insertion was created during an erroneous endogenous repair of the double stranded break performed by Cas9 at the T_4_ position. **(D)** Bar graph representing the average pod length in *wes-arc1* when pollinated with compatible Westar or incompatible *S47* pollen. The error bars represent ±SEM. **(E)** Aniline blue assay to detect pollen attachment and pollen tube penetration in flowers pollinated with pollen from Westar or *S47* haplotypes obtained from *B. rapa*, 24 h post-anthesis. The images were obtained either with UV (280–390 nm) to visualize aniline blue stain or with a green filter to observe pollen attachment. **(F)** Representative picture of pods still attached to inflorescence, pollinated with Westar (self), *S47* (incompatible), and *S8* (compatible), showing the breakdown of SI in the *wes-arc1-1* line. **(G)** Chromatogram generated from Sanger sequencing of *BnARC1* from *W1-arc1-1.* The sequence spans the T_3_ and T_4_ target sites within the *BnARC1* coding region. The red arrows indicate the biallelic insertion of an “A” at precisely 3 bases from the T_4_ PAM sequence and the biallelic insertions of an “A” or “T” at 3 bases from the T_3_ PAM sequence. **(H)** Aniline blue assay to assess pollen attachment and pollen tube penetration in reciprocal crosses of the CRISPR-edited line *W1-arc1-1*, showing breakdown of SI in the absence of *ARC1.* **(I)** Representative picture of mature pods from hand-pollinated individual flowers of the combinations shown. The full extension of pods after incompatible pollination indicates compromised SI in *W1-arc1-1* lines. **(J and K)** Bar graphs representing the average pod length (J) and seed set (K) in *W1-arc1-1* pollinated with W1 or self-pollinated compared with self-pollinated Westar. Error bars represent ±SEM.

We next created a multiplex CRISPR–Cas9 *ARC1* editing construct and generated several *B. napus* Westar (SC) transgenic lines harboring the editing system through *Agrobacterium*-mediated transformation (Ma et al., 2015; Stanic et al., 2021). When *ARC1* was amplified from these transgenic lines, several lines, including the *wes-arc1-1 line*, contained biallelic edits in target 4 that caused a frameshift in the *ARC1* sequence (Figure 1C, only target 3/4 [T_3/4_] shown).

The Westar cultivar is self-compatible due to an insertion in the promoter region of *SP11*, leading to a lack of expression of SP11, the pollen ligand required for SI response (Okamoto et al., 2007). However, it retains all downstream SI signaling components (SRK and ARC1), as it readily rejects pollen of *S47* haplotype origin with a functional SP11 (Okamoto et al., 2007). The T2 generations of two independently edited *wes-arc1* plants with biallelic edits were used for pollination assays with compatible Westar or *S8* pollen and incompatible *S47* pollen. When *B. rapa S47* haplotype pollen was applied to control Westar stigmas, a robust SI was observed in the Westar stigmas, which readily rejected pollen of the *S47* haplotype (Figure 1D–1F). By contrast, the *wes-arc1-1* and *wes-arc1-2* plants showed a complete breakdown of the SI response when pollinated with *S47* pollen (Figure 1D–1F). When either Westar pollen or *B. rapa* pollen from an *S8* haplotype (*SRK8* is absent in Westar) was used as a positive control for stigma receptivity, full acceptance was observed in both Westar controls and *wes-arc1* plants, indicating that stigma receptivity was not modified in the edited line (Figure 1D).

We next sought to test whether nullifying *ARC1* in the well characterized self-incompatible W1 background would result in complete breakdown of SI. The isogenic, self-incompatible W1 line was created by introgression of the dominant 910 *B. rapa* haplotype (SRK 910/SP11-910); it displays a very strong SI phenotype when self-pollinated but is compatible when crossed with Westar. We predicted that regardless of the upstream receptor/ligand complex, abolishing ARC1 function should lead to complete breakdown of SI.

Because W1 plants are incompatible and difficult to propagate/regenerate through conventional *Agrobacterium*-based tissue culture approaches, W1 plants were crossed with the *wes-arc1* mutant transgenic lines harboring the *ARC1* editing system. In the F1 generation, we identified a *W1-arc1-1* line that displayed strong breakdown of SI when self-pollinated. Sequencing of *ARC1* revealed edits at both the T_3/4_ sites (Figure 1G, only T_3/4_ shown). When pollen from *W1-arc1-1* was tested on W1 stigmas, W1 stigmas rejected *W1-arc1-1* pollen, indicating that the *SP11-910* haplotype was not altered or deleted. On the other hand, *ARC1-*edited *W1-arc1-1* plants readily accepted pollen from self-incompatible W1 plants (Figure 1H). This complete breakdown resulted in full seed set (Figure 1I) comparable to that observed when stigmas were pollinated with compatible Westar pollen (Figure 1J and 1K). This compromise in SI was further confirmed in at least four successive generations of *W1-arc1-1* plants that harbored an intact *SRK910*. Flowers from these lines were also used to pollinate W1 stigmas and confirm the SI reaction, demonstrating that the S-haplotype was unmodified in these lines.

We have convincingly demonstrated that elimination of ARC1 results in complete breakdown of SI in two different S-haplotypes (*S47* and *SRK910*), confirming the essential nature of ARC1 for SI response in *Brassica*. In both situations, the closely related PUB17 ortholog was unaltered, indicating the exclusive nature of ARC1 for mediating the SI response. Although this study eliminates any doubt as to whether ARC1 is required for SI in *Brassica*, the fact that ARC1 was shown to be dispensable in certain cases in *A. thaliana* suggests that there could be an evolutionary significance to this observation. In a self-incompatible *A. lyrata* ecotype, ARC1 is present and required for SI response (Indriolo et al., 2012; Indriolo and Goring, 2014), whereas in self-compatible *Arabidopsis* species, *ARC1* is often found to have been deleted (Indriolo et al., 2012, 2014). During the switch from SI to compatibility, *A. thaliana* could have either lost the *ARC1* gene and other components of the SI pathway or neo-functionalized them for various other pathways. Alternatively, ARC1 function could be species specific, as shown in a recent report in which *A. thaliana* SC transgenic lines overexpressing SCR–SRK–ARC1 from *A. halleri* displayed the SI phenotype, whereas they failed to manifest the SI phenotype when the *ARC1* gene was derived from *B. napus*. These results indicate that the display of incompatibility in SC *A. thaliana* might have genus-specific preferences (Zhang et al., 2019).

Nonetheless, this investigation has clearly demonstrated that the absence of functional *BnARC1* in Westar and W1 plants leads to their inability to mount successful SI, showing that ARC1 is an indispensable downstream effector of SI in *Brassica.*

## Funding

This work was supported by the 10.13039/501100000038Natural Sciences and Engineering Research Council of Canada for M.A.S., K.A., and N.M.N.H. X.L. was supported by the 10.13039/501100001809National Natural Science Foundation of China (31870300) and Heilongjiang Touyan Innovation Team Program (Tree Genetics and Breeding Innovation Team).

## Author contributions

M.A.S. and K.A. conceived and designed the experiments. K.A., N.M.N.H., and X.L. performed the experiments. K.A. and M.A.S. analyzed the data. K.A. and M.A.S. wrote the manuscript. M.A.S., K.A., N.M.N.H., and X.L. revised the manuscript. All authors read and approved the final manuscript.

## Supplemental information

Document S1. Table S1 and supplemental materials and methods

Document S2. Article plus supplemental information
