# Supplementary material for: Disabling of ARC1 through CRISPR–Cas9 leads to a complete breakdown of self-incompatibility responses in Brassica napus
Source: Plant Commun. 2022 Dec 14;4(2):100504. doi: 10.1016/j.xplc.2022.100504 (PMC10030360; doi:10.1016/j.xplc.2022.100504)
Supplement: Document S1. Table S1 and supplemental materials and methods [file mmc1.pdf]

Plant Communications, Volume *4*

Supplemental information

Disabling of ARC1 through CRISPR–Cas9 leads to a complete break-down of self-incompatibility responses in *Brassica napus*

Kumar Abhinandan, Neil M.N. Hickerson, Xingguo Lan, and Marcus A. Samuel

Supplemental Information

Disabling of ARC1 through CRISPR/CAS9 leads to a complete breakdown of self-incompatibility responses in *Brassica napus*

Kumar Abhinandan [1,2], Neil M.N. Hickerson[1], Xingguo Lan[3] and Marcus A. Samuel[1]

*Correspondence: msamuel@ucalgary.ca

Affiliations:

[1]University of Calgary, Department of Biological Sciences, 2500 University Drive. NW, Calgary. Alberta T2N 1N4, Calgary, Canada

[2]20/20 Seed Labs Inc., Nisku, Alberta, T9E 7N5, Canada

[3]Key Laboratory of Saline-Alkali Vegetation Ecology Restoration, Ministry of Education, College of Life Sciences, Northeast Forestry University, Harbin 150040, China.

RNA isolation, cDNA synthesis and qPCR of ARC1 and PUB17

Total RNA was isolated from the stigma of *Brassica napus* flowers using TRIzol™ Reagent (Invitrogen™) using the manufacturer's protocol and after quantification and DNase treatment, a total of 2 µg of RNA was aliquoted for cDNA synthesis using the manufacturer's instructions (Applied Biosystems™). One microgram of cDNA was then used for quantitative PCR (qPCR) of the *ARC1* and *PUB17* genes.

CRISPR/Cas9 targeting *BnARC1*: determination of the target site and design of the construct

To generate target sites for the CRISPR/Cas9 system, the CRISPR multi-targeter tool (http://www.multicrispr.net/) was used. This tool uses the input gene sequence to generate target sites that meet the appropriate requirements for Cas9, such as proximity to a protospacer adjacent motif (PAM) site (NGG) (Paul and Qi, 2016). The list of potential gRNA sequences with 45% or higher GC content was selected. It was further narrowed down to find guides with the fewest possible off-targets using the Cas-OFFinder tool (http://www.rgenome.net/cas-offinder/).

The five gRNAs designed to target endogenous *BnARC1* were synthesized into a multiplex construct and cloned into a plasmid that already contained the Cas9 coding sequence under a single CaMV35S promoter control. The five multiplexed gRNAs were each placed its own U3 or U6 promoter. The Cas9 coding sequence was derived from *Streptococcus pyogenes* and optimized for plant codons. This type of multiplexed system was previously shown to be effective in generating double-stranded DNA breaks in canola (Stanic et al., 2021).

In vitro Cas9 digestion of BnARC1 using designed targets

Targets designed for *BnARC1* were tested *in vitro* for their targeting capabilities. The gRNA sequence was amplified using high-fidelity Q5 DNA polymerase from a plasmid containing the guides. Scaffold RNA from the amplicon was then transcribed using a T7 polymerase incubated at 37 °C for 16 hours. The digestion reaction was carried out using purified gRNA, purified Cas9 and *BnARC1* amplicons containing flanking regions of 5' and 3' to allow fragment identification on the gel (Stanic et al., 2021). The Guide-it sgRNA in vitro screening kit (Clontech) was used for Cas9 in vitro digestion. The components were incubated at 37 °C or 1 hour and then heated to 70 °C for 10 minutes for deactivation.

Genetic transformation of Westar and W1 hypocotyls with _Agrobacterium tumefaciens GV3101_

The genetic transformation of canola used here is modified for Westar and W1 transformation (Bhalla and Singh, 2008; Borjian and Arak, 2013). The seeds were sterilized and seeded in round plates (100 x 25) mm containing ½ MS agar (6-7 seeds/plate). After stratification for 2-3 days, the plates were placed in the dark and kept at 22 °C in a vertical position to allow hypocotyl growth. Hypocotyls were cut into smaller pieces of 1 to 1.5 cm and placed on ½ MS agar plates supplemented with 2-4D (1 mg/l) for two days after which *A. tumefaciens GV3101* harboring the construct was used to infect the cut ends and the transgenic plants were regenerated using the procedure described previously (Perkins et al., 2020).

Genomic DNA Isolation

Genomic DNA isolation was performed using chloroform based CTAB method as

previously described (Porebski et al., 1997; Stanic et al., 2021). Leaf tissue was ground in 250 µL CTAB (2% CTAB, 20 mM EDTA, 100 mM Tris-HCl pH 8.0, 1.4 M NaCl + 0.2% β-mercaptoethanol) in microcentrifuge tubes and incubated at 65 °C for 30 - 45 min. Chloroform (250 µL) was added to the pulverized tissues and vortexed for 1 min followed by centrifugation at 12000 rpm for 5 min at RT. The supernatant was transferred to separate tubes without disturbing/aspirating the middle layer. Isopropyl alcohol (150 µL) was added to the tubes and mixed well by vortexing the tubes. Following incubation at -20 °C, the tubes were centrifuged at 12000 rpm for 10 min at RT and all supernatant was discarded. The resulting DNA pellet was washed with 70% ethanol at 12000 rpm for 5 min at RT, followed by drying the pellet for 10 - 30 min. The pellet was resuspended in double-distilled sterile water with brief tapping to dislodge (if any) DNA sticking to the walls of the microfuge tube.

PCR, Genotyping and sequencing of Transgenic Plants for CRISPR/Cas Edits

PCR on isolated plant DNA was performed in a 20 µL final volume using a 2X premix from commercial vendors (VWR, Lambda biotechnology, Thermofisher). The PCR reaction (20 µL) contained 100ng of template DNA; 20pM (0.75 µL of 20 µM) of each forward and reverse primers, 10µL of 2X PCR Master mix. Nano pure water was used to make up the volume to 20µl. PCR reactions were carried out on BioRad S1000™ thermal cycler with the following cycle: 1 denaturation cycle at 95 °C for 3 min, 95 °C for 30 sec, 55 °C for 30 sec, 72 °C for 1 min (based on 1000bp/min extension) (Go to step 2 repeat 30 times), 72 °C for 10 min. The PCR product was analyzed on 1 % agarose gel electrophoresis.

To identify CRISPR edits, BnARC1 was amplified using gene specific primers and edits

were identified using Sanger sequencing.

Table 1: Common Primers Used in Genotyping

Primer	Sequence 5' to 3'
**For CrARC1**	
p1665 For	GCGATTCAAATTCGGCTGACC
Cas9 Seq 379 Rev	TGTCCTCCTCCACGAGGAAGG
**For W1, Westar and Brassica rapa**	
S8-For	TACGTTGGGTATCAGACCGGG
S8-Rev	GACCCCGCGGATATAATGTTTTGC
S47-For	TTAAACGGTAACTCGGGCGAG
S47-Rev	TTCGCATGTCTTGGGTCCGCC
SRK-910-For	CCTACGATAGTTCTTACACT
SRK-910-Rev	CCATGATGTCGGAGTGAACGTT

Aniline Blue Assay

The aniline blue assay was performed as previously described (Samuel et al., 2009). After manually pollinating the stigma of W1, Westar or transgenic plant under investigation, the stigmas were collected after 24 h and fixed in 3:1 ethanol: glacial acetic acid for 1 h to overnight for decolorization. After this, the stigma was gently washed three times with distilled water. The pistils were incubated for 1 h in 1 N NaOH at 60 °C followed by three washes with distilled water and stained for 1 h with basic aniline blue (0.1 M K_3PO_4 0.1% aniline blue) at 60 °C. The stained stigmas were mounted in 50% glycerol, and pollen attachment and pollen germination were observed under the blue channel using a Leica DMR epifluorescence microscope. Attached pollen grains and pollen tubes, if any, were counted and subjected to statistical analysis.

References:

Bhalla, P. L., and Singh, M. B. (2008). Agrobacterium-mediated transformation of Brassica napus and Brassica oleracea. *Nature Protocols* **3**:181–189.

Borjian, L., and Arak, H. (2013). A Study on the Effect of Different Concentration of Plant Hormones (BAP , NAA , 2 , 4-D , and Kinetin) on Callus Induction in Brassica Napus. *Sciences-New York* **5**:519–521.

Ma, X., Zhang, Q., Zhu, Q., Liu, W., Chen, Y., Qiu, R., Wang, B., Yang, Z., Li, H., Lin, Y., et al. (2015). A Robust CRISPR/Cas9 System for Convenient, High-Efficiency Multiplex Genome Editing in Monocot and Dicot Plants. *Molecular Plant* **8**:1274–1284.

Paul, J. W., and Qi, Y. (2016). CRISPR/Cas9 for plant genome editing: accomplishments, problems and prospects. *Plant Cell Reports* **35**:1417–1427.

Perkins, M., Skori, L., Hickerson, N. M. N., Jamshed, M., and Samuel, M. A. (2020). Genetic manipulation of ABI3 confers frost-tolerant seed degreening in canola. *Plant Biotechnology Journal* **18**.

Porebski, S., Bailey, L. G., and Baum, B. R. (1997). Modification of a CTAB DNA extraction protocol for plants containing high polysaccharide and polyphenol components. *Plant Molecular Biology Reporter 1997 15:1* **15**:8–15.

Samuel, M. A., Chong, Y. T., Haasen, K. E., Aldea-Brydges, M. G., Stone, S. L., and Goring, D. R. (2009). Cellular pathways regulating responses to compatible and self-incompatible pollen in brassica and arabidopsis stigmas intersect at exo70a1, a putative component of the exocyst complex. *Plant Cell* **21**:2655–2671.

Stanic, M., Hickerson, N. M. N., Arunraj, R., and Samuel, M. A. (2021). Gene-editing of the strigolactone receptor BnD14 confers promising shoot architectural changes in Brassica napus (canola). *Plant Biotechnology Journal* **19**.
