# Supplementary material for: Disabling of ARC1 through CRISPR–Cas9 leads to a complete breakdown of self-incompatibility responses in Brassica napus
Source: Plant Commun. 2022 Dec 14;4(2):100504. doi: 10.1016/j.xplc.2022.100504 (PMC10030360; doi:10.1016/j.xplc.2022.100504)
Supplement: Document S2. Article plus supplemental information [file mmc2.pdf]

Plant Communications
Correspondence

CellPress
Partner Journal

Disabling of ARC1 through CRISPR–Cas9 leads to a complete breakdown of self-incompatibility responses in *Brassica napus*

Dear Editor,

Many flowering plants utilize the self-incompatibility (SI) response as a genetic mechanism to prevent self-pollen from establishing on the stigmas, thereby promoting outcrossing and genetic diversity. In *Brassica* during SI, recognition of the pollen ligand SP11 by S-locus receptor kinase (SRK) results in activation of the E3 ligase ARM-Repeat-Containing protein (ARC1), which leads to proteasomal degradation of compatibility factors required for successful pollen acceptance. ARC1 was originally identified as an interactor of the SRK kinase domain and is highly expressed in mature stigmas (Gu et al., 1998). Antisense suppression of ARC1 resulted in partial breakdown of SI in the self-incompatible *Brassica napus* W1 line, establishing the role of ARC1 as a positive regulator of SI (Stone et al., 1999). This was further supported by RNAi-mediated suppression of ARC1 in the self-incompatible *A. lyrata*, which resulted in partial breakdown of the SI pathway (Indriolo et al., 2012). The observed partial compromise of SI in both *Brassica* and *Arabidopsis* suggested that either an alternative SI pathway or incomplete suppression of ARC1 could have resulted in the incomplete breakdown of SI. This question has remained unresolved. In this study, we created *ARC1* loss-of-function *B. napus* lines to demonstrate the central role of ARC1 in mediating the SI response.

Despite the partial breakdown of SI shown in two different systems, the role of ARC1 in the Brassicaceae SI response has been questioned for the past several decades. Stable co-expression of *Brassica SLG*, *SRK*, and *ARC1* was insufficient to confer the SI phenotype in compatible *Arabidopsis thaliana* (Bi et al., 2000). In another report, when *A. thaliana* plants were transformed to express SRK_b and SCR_b genes, a strong SI phenotype was observed in the absence of ARC1 (Kitashiba et al., 2011). *A. thaliana* plants that exhibit the SI phenotype through transformation of *SRK* and *SCR* alone show trends in heritability, developmental regulation, and intensity of SI response similar to those of self-incompatible *Brassica* cultivars (Nasrallah and Nasrallah, 2014). Although, evolutionarily, *Arabidopsis* that lacked a *bona fide* ARC1 ortholog could have re-purposed other E3 ligases to assume the role of ARC1, the question of how complete loss of ARC1 could influence SI in *Brassica* sp. remained unresolved.

To unequivocally examine the role of ARC1 during SI, we created *ARC1* loss-of-function *B. napus* using the CRISPR–Cas9 platform. In order to efficiently design specific targets for the CRISPR constructs, we retrieved available gene copies of *ARC1* or similar genes present in *B. napus* (allotetraploid from the A and C genomes of *B. rapa* and *B.* oleracea) (http://cbi.hzau.edu.cn/bnapus/index.php) (Song et al., 2020). After validation of the sequences across the published NCBI database, TOPO cloning was performed with *BnARC1*-specific cDNA amplification products obtained from an RNA pool of fully mature *B. napus* stigmas. Sanger sequencing of the clones revealed that *BnARC1* is a single-copy gene derived from *Brassica rapa* (A genome of *rapa*), whereas its homolog from the C genome (oleracea) was identified at low frequency and was most similar to *PUB17* family genes (Figure 1A). When the expression profiles of these two genes were assessed at various stages of stigma maturity, *ARC1* had significantly higher expression than *PUB17* and peaked at stigma maturity (Figure 1B).

We next created a multiplex CRISPR–Cas9 *ARC1* editing construct and generated several *B. napus* Westar (SC) transgenic lines harboring the editing system through *Agrobacterium*-mediated transformation (Ma et al., 2015; Stanic et al., 2021). When *ARC1* was amplified from these transgenic lines, several lines, including the *wes-arc1-1 line*, contained biallelic edits in target 4 that caused a frameshift in the *ARC1* sequence (Figure 1C, only target 3/4 [$T_{3/4}$] shown).

The Westar cultivar is self-compatible due to an insertion in the promoter region of *SP11*, leading to a lack of expression of SP11, the pollen ligand required for SI response (Okamoto et al., 2007). However, it retains all downstream SI signaling components (SRK and ARC1), as it readily rejects pollen of *S47* haplotype origin with a functional SP11 (Okamoto et al., 2007). The T2 generations of two independently edited *wes-arc1* plants with biallelic edits were used for pollination assays with compatible Westar or *S8* pollen and incompatible *S47* pollen. When *B. rapa S47* haplotype pollen was applied to control Westar stigmas, a robust SI was observed in the Westar stigmas, which readily rejected pollen of the *S47* haplotype (Figure 1D–1F). By contrast, the *wes-arc1-1* and *wes-arc1-2* plants showed a complete breakdown of the SI response when pollinated with *S47* pollen (Figure 1D–1F). When either Westar pollen or *B. rapa* pollen from an *S8* haplotype (*SRK8* is absent in Westar) was used as a positive control for stigma receptivity, full acceptance was observed in both Westar controls and *wes-arc1* plants, indicating that stigma receptivity was not modified in the edited line (Figure 1D).

We next sought to test whether nullifying *ARC1* in the well characterized self-incompatible W1 background would result in

Published by the Plant Communications Shanghai Editorial Office in association with Cell Press, an imprint of Elsevier Inc., on behalf of CSPB and CEMPS, CAS.

This is an open access article under the CC BY license (http://creativecommons.org/licenses/by/4.0/).

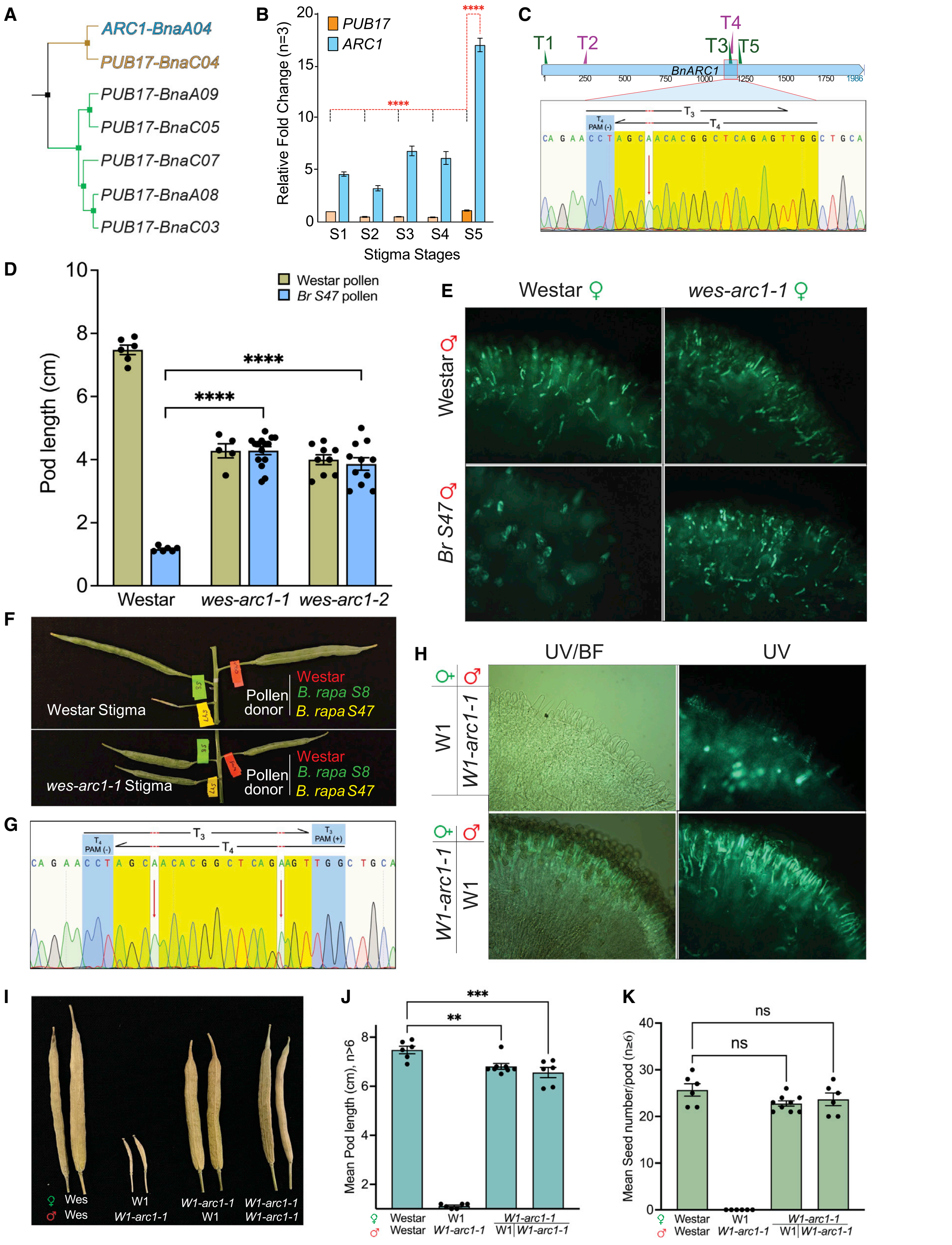

Figure 1. CRISPR–CAS9-mediated editing of *ARM-Repeat-Containing 1* protein (*ARC1*) leads to a complete breakdown of the self-incompatibility response in *Brassica napus*.

(A) *ARC1* and related sequences were retrieved from the *Brassica napus pangenome information resource* database. Highly divergent *PUB17* (*PUB17-BnaC03a*) is not shown in the cladogram.

(legend continued on next page)

complete breakdown of SI. The isogenic, self-incompatible W1 line was created by introgression of the dominant 910 *B. rapa* haplotype (SRK 910/SP11-910); it displays a very strong SI phenotype when self-pollinated but is compatible when crossed with Westar. We predicted that regardless of the upstream receptor/ligand complex, abolishing ARC1 function should lead to complete breakdown of SI.

Because W1 plants are incompatible and difficult to propagate/regenerate through conventional *Agrobacterium*-based tissue culture approaches, W1 plants were crossed with the *wes-arc1* mutant transgenic lines harboring the *ARC1* editing system. In the F1 generation, we identified a *W1-arc1-1* line that displayed strong breakdown of SI when self-pollinated. Sequencing of *ARC1* revealed edits at both the $T_{3/4}$ sites (Figure 1G, only $T_{3/4}$ shown). When pollen from *W1-arc1-1* was tested on W1 stigmas, W1 stigmas rejected *W1-arc1-1* pollen, indicating that the *SP11-910* haplotype was not altered or deleted. On the other hand, *ARC1*-edited *W1-arc1-1* plants readily accepted pollen from self-incompatible W1 plants (Figure 1H). This complete breakdown resulted in full seed set (Figure 1I) comparable to that observed when stigmas were pollinated with compatible Westar pollen (Figure 1J and 1K). This compromise in SI was further confirmed in at least four successive generations of *W1-arc1-1* plants that harbored an intact *SRK910*. Flowers from these lines were also used to pollinate W1 stigmas and confirm the SI reaction, demonstrating that the S-haplotype was unmodified in these lines.

We have convincingly demonstrated that elimination of ARC1 results in complete breakdown of SI in two different S-haplotypes (*S47* and *SRK910*), confirming the essential nature of ARC1 for SI response in *Brassica*. In both situations, the closely related PUB17 ortholog was unaltered, indicating the exclusive nature of ARC1 for mediating the SI response. Although this study eliminates any doubt as to whether ARC1 is required for SI in *Brassica*, the fact that ARC1 was shown to be dispensable in certain cases in *A. thaliana* suggests that there could be an evolutionary significance to this observation. In a self-incompatible *A. lyrata* ecotype, ARC1 is present and required for SI response (Indriolo et al., 2012; Indriolo and Goring, 2014), whereas in self-compatible *Arabidopsis* species, *ARC1* is often found to have been deleted (Indriolo et al., 2012, 2014). During the switch from SI to compatibility, *A. thaliana* could have either lost the *ARC1* gene and other components of the SI pathway or neo-functionalized them for various other pathways. Alternatively, ARC1 function could be species specific, as shown in a recent report in which *A. thaliana* SC transgenic lines overexpressing SCR–SRK–ARC1 from *A. halleri* displayed the SI phenotype, whereas they failed to manifest the SI phenotype when the *ARC1* gene was derived from *B. napus*. These results indicate that the display of incompatibility in SC *A. thaliana* might have genus-specific preferences (Zhang et al., 2019).

Nonetheless, this investigation has clearly demonstrated that the absence of functional *BnARC1* in Westar and W1 plants leads to their inability to mount successful SI, showing that ARC1 is an indispensable downstream effector of SI in *Brassica.*

SUPPLEMENTAL INFORMATION

Supplemental information is available at *Plant Communications Online*.

FUNDING

This work was supported by the Natural Sciences and Engineering Research Council of Canada for M.A.S., K.A., and N.M.N.H. X.L. was supported by the National Natural Science Foundation of China (31870300) and Heilongjiang Touyan Innovation Team Program (Tree Genetics and Breeding Innovation Team).

AUTHOR CONTRIBUTIONS

M.A.S. and K.A. conceived and designed the experiments. K.A., N.M.N.H., and X.L. performed the experiments. K.A. and M.A.S. analyzed the data. K.A. and M.A.S. wrote the manuscript. M.A.S., K.A., N.M.N.H., and X.L. revised the manuscript. All authors read and approved the final manuscript.

ACKNOWLEDGMENTS

No conflict of interest is declared.

(B) Relative expression of *ARC1* and *PUB17* during stigma development was assessed by qPCR. The bars show the fold change in *BnPUB17* and *BnARC1* during stigma development. Error bars represent the standard error of the mean (±SEM).

(C) Schematic diagram showing the location of the various guideRNA sites along with the chromatogram generated from Sanger sequencing of *BnARC1* from *wes-arc1-1.* Sequence spans the T_3 and T_4 target sites within the *BnARC1* coding region. The red arrow indicates the biallelic insertion of an “A” at precisely 3 bases from the T_4 protospacer adjacent motif (PAM) sequence, indicating that the insertion was created during an erroneous endogenous repair of the double stranded break performed by Cas9 at the T_4 position.

(D) Bar graph representing the average pod length in *wes-arc1* when pollinated with compatible Westar or incompatible *S47* pollen. The error bars represent ±SEM.

(E) Aniline blue assay to detect pollen attachment and pollen tube penetration in flowers pollinated with pollen from Westar or *S47* haplotypes obtained from *B. rapa*, 24 h post-anthesis. The images were obtained either with UV (280–390 nm) to visualize aniline blue stain or with a green filter to observe pollen attachment.

(F) Representative picture of pods still attached to inflorescence, pollinated with Westar (self), *S47* (incompatible), and *S8* (compatible), showing the breakdown of SI in the *wes-arc1-1* line.

(G) Chromatogram generated from Sanger sequencing of *BnARC1* from *W1-arc1-1.* The sequence spans the T_3 and T_4 target sites within the *BnARC1* coding region. The red arrows indicate the biallelic insertion of an “A” at precisely 3 bases from the T_4 PAM sequence and the biallelic insertions of an “A” or “T” at 3 bases from the T_3 PAM sequence.

(H) Aniline blue assay to assess pollen attachment and pollen tube penetration in reciprocal crosses of the CRISPR-edited line *W1-arc1-1*, showing breakdown of SI in the absence of *ARC1.*

(I) Representative picture of mature pods from hand-pollinated individual flowers of the combinations shown. The full extension of pods after incompatible pollination indicates compromised SI in *W1-arc1-1* lines.

(J and K) Bar graphs representing the average pod length (J) and seed set (K) in *W1-arc1-1* pollinated with W1 or self-pollinated compared with self-pollinated Westar. Error bars represent ±SEM.

Received: August 8, 2022
Revised: November 9, 2022
Accepted: December 12, 2022
Published: December 14, 2022

Kumar Abhinandan[1,2], *Neil M.N. Hickerson*[1], *Xingguo Lan*[3] *and Marcus A. Samuel*[1,*]

[1]University of Calgary, Department of Biological Sciences, 2500 University Drive NW, Calgary, AB T2N 1N4, Canada
[2]20/20 Seed Labs Inc., Nisku, AB T9E 7N5, Canada
[3]Key Laboratory of Saline-Alkali Vegetation Ecology Restoration, Ministry of Education, College of Life Sciences, Northeast Forestry University, Harbin 150040, China
***Correspondence: Marcus A. Samuel (msamuel@ucalgary.ca)**
https://doi.org/10.1016/j.xplc.2022.100504

REFERENCES

Bi, Y.M., Brugière, N., Cui, Y., Goring, D.R., and Rothstein, S.J. (2000). Transformation of Arabidopsis with a brassica SLG/SRK region and ARC1 gene is not sufficient to transfer the self-incompatibility phenotype. Mol. Gen. Genet. **263**:648–654.

Gu, T., Mazzurco, M., Sulaman, W., Matias, D.D., and Goring, D.R. (1998). Binding of an arm repeat protein to the kinase domain of the S-locus receptor kinase. Proc. Natl. Acad. Sci. USA **95**:382–387.

Indriolo, E., and Goring, D.R. (2014). A conserved role for the ARC1 E3 ligase in Brassicaceae self-incompatibility. Front. Plant Sci. **5**:181.

Indriolo, E., Tharmapalan, P., Wright, S.I., and Goring, D.R. (2012). The ARC1 E3 ligase gene is frequently deleted in self-compatible brassicaceae species and has a conserved role in arabidopsis lyrata self-pollen rejectionw. Plant Cell **24**:4607–4620.

Indriolo, E., Safavian, D., and Goring, D.R. (2014). The ARC1 E3 ligase promotes two different self-pollen avoidance traits in Arabidopsis. Plant Cell **26**:1525–1543.

Kitashiba, H., Liu, P., Nishio, T., Nasrallah, J.B., and Nasrallah, M.E. (2011). Functional test of Brassica self-incompatibility modifiers in Arabidopsis thaliana. Proc. Natl. Acad. Sci. USA **108**:18173–18178.

Ma, X., Zhang, Q., Zhu, Q., Liu, W., Chen, Y., Qiu, R., Wang, B., Yang, Z., Li, H., Lin, Y., et al. (2015). A robust CRISPR/Cas9 system for convenient, high-efficiency multiplex genome editing in monocot and dicot plants. Mol. Plant **8**:1274–1284.

Nasrallah, J.B., and Nasrallah, M.E. (2014). Robust self-incompatibility in the absence of a functional ARC1 gene in Arabidopsis thaliana. Plant Cell **26**:3838–3841.

Okamoto, S., Odashima, M., Fujimoto, R., Sato, Y., Kitashiba, H., and Nishio, T. (2007). Self-compatibility in Brassica napus is caused by independent mutations in S-locus genes. Plant J. **50**:391–400.

Song, J.M., Liu, D.X., Xie, W.Z., Yang, Z., Guo, L., Liu, K., Yang, Q.Y., and Chen, L.L. (2020). BnPIR: Brassica napus Pan-genome Information Resource for 1, 689 accessions. Plant Biotechnol. J. **19**:412–414. https://doi.org/10.1111/pbi.13491.

Stanic, M., Hickerson, N.M.N., Arunraj, R., and Samuel, M.A. (2021). Gene-editing of the strigolactone receptor BnD14 confers promising shoot architectural changes in Brassica napus (canola). Plant Biotechnol. J. **19**:639–641.

Stone, S.L., Arnoldo, M., and Goring, D.R. (1999). A breakdown of Brassica self-incompatibility in ARC1 antisense transgenic plants. Science **286**:1729–1731.

Zhang, T., Zhou, G., Goring, D.R., Liang, X., Macgregor, S., Dai, C., Wen, J., Yi, B., Shen, J., Tu, J., et al. (2019). Generation of transgenic self-incompatible Arabidopsis thaliana shows a genus-specific preference for self-incompatibility genes. Plants **8**:570–616.

 © 2022 The Authors.

Plant Communications, Volume *4*

Supplemental information

Disabling of ARC1 through CRISPR–Cas9 leads to a complete break-down of self-incompatibility responses in *Brassica napus*

Kumar Abhinandan, Neil M.N. Hickerson, Xingguo Lan, and Marcus A. Samuel

Supplemental Information

Disabling of ARC1 through CRISPR/CAS9 leads to a complete breakdown of self-incompatibility responses in *Brassica napus*

Kumar Abhinandan [1,2], Neil M.N. Hickerson[1], Xingguo Lan[3] and Marcus A. Samuel[1]

*Correspondence: msamuel@ucalgary.ca

Affiliations:

[1]University of Calgary, Department of Biological Sciences, 2500 University Drive. NW, Calgary. Alberta T2N 1N4, Calgary, Canada

[2]20/20 Seed Labs Inc., Nisku, Alberta, T9E 7N5, Canada

[3]Key Laboratory of Saline-Alkali Vegetation Ecology Restoration, Ministry of Education, College of Life Sciences, Northeast Forestry University, Harbin 150040, China.

RNA isolation, cDNA synthesis and qPCR of ARC1 and PUB17

Total RNA was isolated from the stigma of *Brassica napus* flowers using TRIzol™ Reagent (Invitrogen™) using the manufacturer's protocol and after quantification and DNase treatment, a total of 2 µg of RNA was aliquoted for cDNA synthesis using the manufacturer's instructions (Applied Biosystems™). One microgram of cDNA was then used for quantitative PCR (qPCR) of the *ARC1* and *PUB17* genes.

CRISPR/Cas9 targeting *BnARC1*: determination of the target site and design of the construct

To generate target sites for the CRISPR/Cas9 system, the CRISPR multi-targeter tool (http://www.multicrispr.net/) was used. This tool uses the input gene sequence to generate target sites that meet the appropriate requirements for Cas9, such as proximity to a protospacer adjacent motif (PAM) site (NGG) (Paul and Qi, 2016). The list of potential gRNA sequences with 45% or higher GC content was selected. It was further narrowed down to find guides with the fewest possible off-targets using the Cas-OFFinder tool (http://www.rgenome.net/cas-offinder/).

The five gRNAs designed to target endogenous *BnARC1* were synthesized into a multiplex construct and cloned into a plasmid that already contained the Cas9 coding sequence under a single CaMV35S promoter control. The five multiplexed gRNAs were each placed its own U3 or U6 promoter. The Cas9 coding sequence was derived from *Streptococcus pyogenes* and optimized for plant codons. This type of multiplexed system was previously shown to be effective in generating double-stranded DNA breaks in canola (Stanic et al., 2021).

In vitro Cas9 digestion of BnARC1 using designed targets

Targets designed for *BnARC1* were tested *in vitro* for their targeting capabilities. The gRNA sequence was amplified using high-fidelity Q5 DNA polymerase from a plasmid containing the guides. Scaffold RNA from the amplicon was then transcribed using a T7 polymerase incubated at 37 °C for 16 hours. The digestion reaction was carried out using purified gRNA, purified Cas9 and *BnARC1* amplicons containing flanking regions of 5' and 3' to allow fragment identification on the gel (Stanic et al., 2021). The Guide-it sgRNA in vitro screening kit (Clontech) was used for Cas9 in vitro digestion. The components were incubated at 37 °C or 1 hour and then heated to 70 °C for 10 minutes for deactivation.

Genetic transformation of Westar and W1 hypocotyls with _Agrobacterium tumefaciens GV3101_

The genetic transformation of canola used here is modified for Westar and W1 transformation (Bhalla and Singh, 2008; Borjian and Arak, 2013). The seeds were sterilized and seeded in round plates (100 x 25) mm containing ½ MS agar (6-7 seeds/plate). After stratification for 2-3 days, the plates were placed in the dark and kept at 22 °C in a vertical position to allow hypocotyl growth. Hypocotyls were cut into smaller pieces of 1 to 1.5 cm and placed on ½ MS agar plates supplemented with 2-4D (1 mg/l) for two days after which *A. tumefaciens GV3101* harboring the construct was used to infect the cut ends and the transgenic plants were regenerated using the procedure described previously (Perkins et al., 2020).

Genomic DNA Isolation

Genomic DNA isolation was performed using chloroform based CTAB method as

previously described (Porebski et al., 1997; Stanic et al., 2021). Leaf tissue was ground in 250 µL CTAB (2% CTAB, 20 mM EDTA, 100 mM Tris-HCl pH 8.0, 1.4 M NaCl + 0.2% β-mercaptoethanol) in microcentrifuge tubes and incubated at 65 °C for 30 - 45 min. Chloroform (250 µL) was added to the pulverized tissues and vortexed for 1 min followed by centrifugation at 12000 rpm for 5 min at RT. The supernatant was transferred to separate tubes without disturbing/aspirating the middle layer. Isopropyl alcohol (150 µL) was added to the tubes and mixed well by vortexing the tubes. Following incubation at -20 °C, the tubes were centrifuged at 12000 rpm for 10 min at RT and all supernatant was discarded. The resulting DNA pellet was washed with 70% ethanol at 12000 rpm for 5 min at RT, followed by drying the pellet for 10 - 30 min. The pellet was resuspended in double-distilled sterile water with brief tapping to dislodge (if any) DNA sticking to the walls of the microfuge tube.

PCR, Genotyping and sequencing of Transgenic Plants for CRISPR/Cas Edits

PCR on isolated plant DNA was performed in a 20 µL final volume using a 2X premix from commercial vendors (VWR, Lambda biotechnology, Thermofisher). The PCR reaction (20 µL) contained 100ng of template DNA; 20pM (0.75 µL of 20 µM) of each forward and reverse primers, 10µL of 2X PCR Master mix. Nano pure water was used to make up the volume to 20µl. PCR reactions were carried out on BioRad S1000™ thermal cycler with the following cycle: 1 denaturation cycle at 95 °C for 3 min, 95 °C for 30 sec, 55 °C for 30 sec, 72 °C for 1 min (based on 1000bp/min extension) (Go to step 2 repeat 30 times), 72 °C for 10 min. The PCR product was analyzed on 1 % agarose gel electrophoresis.

To identify CRISPR edits, BnARC1 was amplified using gene specific primers and edits

were identified using Sanger sequencing.

Table 1: Common Primers Used in Genotyping

Primer	Sequence 5’ to 3’
**For CrARC1**	
p1665 For	GCGATTCAAATTCGGCTGACC
Cas9 Seq 379 Rev	TGTCCTCCTCCACGAGGAAGG
**For W1, Westar and Brassica rapa**	
S8-For	TACGTTGGGTATCAGACCGGG
S8-Rev	GACCCCGCGGATATAATGTTTTGC
S47-For	TTAAACGGTAACTCGGGCGAG
S47-Rev	TTCGCATGTCTTGGGTCCGCC
SRK-910-For	CCTACGATAGTTCTTACACT
SRK-910-Rev	CCATGATGTCGGAGTGAACGTT

Aniline Blue Assay

The aniline blue assay was performed as previously described (Samuel et al., 2009). After manually pollinating the stigma of W1, Westar or transgenic plant under investigation, the stigmas were collected after 24 h and fixed in 3:1 ethanol: glacial acetic acid for 1 h to overnight for decolorization. After this, the stigma was gently washed three times with distilled water. The pistils were incubated for 1 h in 1 N NaOH at 60 °C followed by three washes with distilled water and stained for 1 h with basic aniline blue (0.1 M K_3PO_4 0.1% aniline blue) at 60 °C. The stained stigmas were mounted in 50% glycerol, and pollen attachment and pollen germination were observed under the blue channel using a Leica DMR epifluorescence microscope. Attached pollen grains and pollen tubes, if any, were counted and subjected to statistical analysis.

References:

Bhalla, P. L., and Singh, M. B. (2008). Agrobacterium-mediated transformation of Brassica napus and Brassica oleracea. *Nature Protocols* **3**:181–189.

Borjian, L., and Arak, H. (2013). A Study on the Effect of Different Concentration of Plant Hormones (BAP , NAA , 2 , 4-D , and Kinetin) on Callus Induction in Brassica Napus. *Sciences-New York* **5**:519–521.

Ma, X., Zhang, Q., Zhu, Q., Liu, W., Chen, Y., Qiu, R., Wang, B., Yang, Z., Li, H., Lin, Y., et al. (2015). A Robust CRISPR/Cas9 System for Convenient, High-Efficiency Multiplex Genome Editing in Monocot and Dicot Plants. *Molecular Plant* **8**:1274–1284.

Paul, J. W., and Qi, Y. (2016). CRISPR/Cas9 for plant genome editing: accomplishments, problems and prospects. *Plant Cell Reports* **35**:1417–1427.

Perkins, M., Skori, L., Hickerson, N. M. N., Jamshed, M., and Samuel, M. A. (2020). Genetic manipulation of ABI3 confers frost-tolerant seed degreening in canola. *Plant Biotechnology Journal* **18**.

Porebski, S., Bailey, L. G., and Baum, B. R. (1997). Modification of a CTAB DNA extraction protocol for plants containing high polysaccharide and polyphenol components. *Plant Molecular Biology Reporter 1997 15:1* **15**:8–15.

Samuel, M. A., Chong, Y. T., Haasen, K. E., Aldea-Brydges, M. G., Stone, S. L., and Goring, D. R. (2009). Cellular pathways regulating responses to compatible and self-incompatible pollen in brassica and arabidopsis stigmas intersect at exo70a1, a putative component of the exocyst complex. *Plant Cell* **21**:2655–2671.

Stanic, M., Hickerson, N. M. N., Arunraj, R., and Samuel, M. A. (2021). Gene-editing of the strigolactone receptor BnD14 confers promising shoot architectural changes in Brassica napus (canola). *Plant Biotechnology Journal* **19**.
